# Full Transcriptome Analysis of Callus Suspension Culture System of *Bletilla striata*

**DOI:** 10.3389/fgene.2020.00995

**Published:** 2020-10-15

**Authors:** Lin Li, Houbo Liu, Weie Wen, Ceyin Huang, Xiaomei Li, Shiji Xiao, Mingkai Wu, Junhua Shi, Delin Xu

**Affiliations:** ^1^Department of Cell Biology, Zunyi Medical University, Zunyi, China; ^2^School of Pharmacy, Zunyi Medical University, Zunyi, China; ^3^Institute of Modern Chinese Herbal of Guizhou Academy of Agricultural Sciences, Guiyang, China; ^4^The Department of Imaging, Affiliated Hospital of Zunyi Medical University, Zunyi, China

**Keywords:** *Bletilla striata*, suspension culture, transcriptome sequencing, functional annotation, lncRNA, SSR

## Abstract

**Background:**

*Bletilla striata* has been widely used in the pharmacology industry. To effectively produce the secondary metabolites through suspension cultured cells of *B. striata*, it is important to exploring the full-length transcriptome data and the genes related to cell growth and chemical producing of all culture stages. We applied a combination of Real-Time Sequencing of Single Molecule (SMRT) and second-generation sequencing (SGS) to generate the complete and full-length transcriptome of *B. striata* suspension cultured cells.

**Methods:**

The *B. striata* transcriptome was formed in *de novo* way by using PacBio isoform sequencing (Iso-Seq) on a pooled RNA sample derived from 23 samples of 10 culture stages, to explore the potential for capturing full-length transcript isoforms. All unigenes were obtained after splicing, assembling, and clustering, and corrected by the SGS results. The obtained unigenes were compared with the databases, and the functions were annotated and classified.

**Results and conclusions:**

A total of 100,276 high-quality full-length transcripts were obtained, with an average length of 2530 bp and an N50 of 3302 bp. About 52% of total sequences were annotated against the Gene Ontology, 53,316 unigenes were hit by KOG annotations and divided into 26 functional categories, 80,020 unigenes were mapped by KEGG annotations and clustered into 363 pathways. Furthermore, 15,133 long-chain non-coding RNAs (lncRNAs) were detected. And 68,996 coding sequences were identified based on SSR analysis, among which 31 pairs of primers selected at random were amplified and obtained stable bands. In conclusion, our results provide new full-length transcriptome data and genetic resources for identifying growth and metabolism-related genes, which provide a solid foundation for further research on its growth regulation mechanisms and genetic engineering breeding mechanisms of *B. striata*.

## Introduction

*Bletilla striata* is a traditional Chinese medicinal (TCM) herb, spelled in Chinese as “Baiji,” which is widely used in the treatment of pulmonary and gastric diseases such as pulmonary and gastric bleeding, silicosis, tuberculosis, gastric ulcers, etc. (Xu et al., 2019). It can also be used with other TCM to treat skin cracks, burns, freckles, etc. (Liao et al., 2018; Jiang S. et al., 2019). It is a traditional and precious TCM in China, which was first recorded in Shennong’s Materia Medica (Shen Nong Ben Cao Jing) and has been used for more than two thousand years (Xu et al., 2019). In recent years, increasing studies have found that a variety of secondary metabolite components in *B. striata* have plentiful pharmacological activities, such as anti-influenza virus, anti-tumor, and protection of gastric mucosa (Wang and Meng, 2015; Lin et al., 2016; Jiang F. S. et al., 2019). In addition, *B. striata* not only has outstanding medicinal value, it is also an important ornamental plant in the United States, China, Europe and some other countries (Xu et al., 2019).

With the vigorous development of TCM in recent years and the increasing market demand for Chinese medicine, the development and utilization of TCM resources have continued to heat up, bringing unprecedented opportunities and challenges to its industry in China (Wang et al., 2014). Active medicinal ingredients are the material basis of TCM. It is necessary to study how to efficiently synthesize the main medicinal ingredients in herbal medicine. At present, researches on the use of synthetic biology in the sustainable use of TCM resources have received widespread attention. Synthetic biology is an emerging discipline that analyzes the biosynthetic pathways of active ingredients of TCM and discovers functional genes involved in biosynthesis. Thus, synthetic biology will play an indispensable role in the approaching of modernization and globalization of Chinese medicinal materials by virtue of its advantages. As for *B. striata*, however, due to the lack of genetic information for the whole process of cell growth and synthetic pathways of secondary metabolites, its progress in this field lags far behind other plants. Therefore, we have established a nucleotide database of full-length transcripts profiled throughout the whole growing period of the culturing system, which will facilitate the exploration of the molecular mechanisms of cell proliferation and metabolites synthesis. Meanwhile, it is also significant to rely on the use of suspension cell culture technology for cultivation and breeding combined with the study of secondary metabolic pathways and their key enzyme functional genes to improve production and pharmacological applications of *B. striata*.

The combination of conventional breeding and molecular biology is a promising way for plant genetic improvement. With the advent of the post-genome era, transcriptome, proteomics, metabonomics and other technologies have emerged one after another, among which transcriptome is the first to develop and the most widely used (Besser et al., 2018; Ghosh et al., 2018; Kumar et al., 2019). In recent years, with the development of high-throughput sequencing technology, transcriptome sequencing has become an important means to study the regulation of gene expression (Wang et al., 2009). It is the basis and starting point for studying the function and structure of genes, and plays a very important role in understanding development and diseases resistance. However, due to the limitation of the lengthy reading of the second-generation sequencing, the full-length transcript obtained by its splicing is not complete, and the third-generation sequencing technology represented by PacBio has solved this problem effectively. The platform uses single molecule real-time sequencing technology, also known as SMRT sequencing, with the advantage of ultra-long reading length, high-quality full-length transcript information can be obtained directly, avoiding the assembly (Li Y. P. et al., 2017). At present, three *B. striata* databases have been found on NCBI, but most of the unigenes are non-full-length sequences. Our group constructed the first nucleotide database before (Xu et al., 2018), but because of the spatio-temporal specificity and conditions of gene expression, the previous database is not suitable for gene mining and mechanism analysis under the condition of suspension culture. Therefore, it is essential to establish a comprehensively annotated database consisting of full-length transcripts which profile throughout the whole growing period of callus suspension culturing system, obtained by combining the second-generation sequencing technology with the third generation sequencing technology.

According to the published literature, there have been a number of reports on transcriptome analysis based on PacBio platform. For example, Jia used SMRT sequencing technology to obtain the full-length transcript data of flea beetle *Agasicles hygrophila*, in which 28,982 transcripts were obtained in which 24,031 transcripts were annotated in eight functional databases (Jia et al., 2018). Zhang used SMRT technology to analyze the whole genome of cis natural antisense transcripts (cis-NATs) in *Phyllostachys pubescens* for the first time who found a total of 932 cis-NATs and 22196 alternative splicing (AS) events, in which 42 cis-NATs and 442 AS events were differentially expressed when exposed to foreign GA3, indicating that post-transcriptional regulation may also be involved in GA3 response (Zhang et al., 2018). Hoang conducted a full-length transcriptome analysis of sugarcane (*Saccharum* L.) by using Iso-Seq technology, a total of 107,598 transcripts were obtained, in which 92% of the data set matched the plant protein database and total of 4,870 variable splicing events were detected by TAPIS, including 1,302 intron retention, 559 exon jumps, 1,365 5′ end variable splicing and 1,644 3′ segment variable splicing (Hoang et al., 2017). However, there is no report related to the full-length transcriptional sequencing in *B. striata*, let alone one about the suspension system.

Previously, our research on *B. striata* transcriptome relied on next-generation sequencing (NGS) technology, and most of these studies targeted whole plants. Therefore, in order to enrich the genetic information of suspension system and improve the overall accuracy of *B. striata* gene prediction, in this study, we combined with two sequencing technologies of NGS and PacBio SMRT to construct a complete full-length transcript database. To obtain this, 23 samples of *B. striata* suspension cultured cells were extracted from 10 different time points. By analyzing the levels of transcriptomes from different treatments, we provided a theoretical basis for finding out the key genes that are involved in cell growth and chemical producing, and further exploring the transcriptional regulation mechanism at the overall transcription level. This research offered some new information to explain the growth regulation mechanism and genetic engineering breeding mechanism of *B. striata*.

## Materials and Methods

### Callus Suspension Culture and Growth Curve Drawing

#### Callus Induction

The matured capsules of *B. striata* collected from Zhengan County, Guizhou Province, China (28°56′N, 107°43′E) were used for callus inducing. Capsules were first rinsed 5 mins under running water, then with ultra-pure water, placed on a super purification table, and disinfected it with 0.1 liter mercury dichloride for 5 min, then repeatedly rinsed with sterile water 3 times, and dried the surface moisture with sterile paper. Secondly, the inside seeds of capsules were gently clamped and evenly sowed on the surface of the induction medium and further placed at 25°C for dark culture. The formula of induction medium was MS + 1.0 mg/L 6-BA + 3.0 mg/L 2,4-D + 30.0 g/L sucrose + 10.0 g/L Agar.

#### Subculture of Callus

When the seeds were induced to granular callus, subculture was carried out. After about 45 days, the calluses with diameters of 0.15 cm were transferred into the corresponding subculture medium at 25°C in the dark. The formula of subculture medium was MS + 1.0 mg/L 6-BA + 2.0 mg/L 2,4-D + 30.0 g/L sucrose + 10.0 g/L Agar.

#### Suspension Culture System

The culture system was improved based on our former study (Pan et al., 2020). The loose and bright yellow callus obtained after two subculture processes for about 30 days were used as explants for suspension culturing and inoculating into 100 mL culture flask which containing 35 mL liquid medium. The pH was 5.95 and the inoculation amount of explant was 1.0 g (fresh weight) per bottle. After inoculation, the culture was placed in a rotary shaker with a speed of 120 rpm, temperature of 25°C and in the dark. The formula of the culture medium was as follows: MS + 1.0 mg/L 6-BA + 3.0 mg/L 2,4-D + 30.0 g/L sucrose.

#### Weight Determination of Fresh and Dry Callus

Under the above culture conditions, three samples were randomly sampled at every 3 days from the first inoculation day (0 dpi) to 45 dpi for measuring the fresh weight and dry weight of culturing callus. The liquid in the culture bottle was extracted and filtered, and the obtained callus was measured as fresh weight. Then, it was baked in a 50°C oven until the constant and weighted as dry weight.

### RNA Sample Preparation

The calluses grown in the suspension system were randomly sampled every 3 days. For each timepoint, 3 duplicates were taken. An appropriate amount of each sample were quickly grinded with liquid nitrogen for extracting total RNA by Trizol solution and then stored in refrigerator at −80°C for further conducts.

### Library Preparation, Illumina and SMRT Sequencing

The RNA samples were entrusted to Novogene Co., Ltd., for library construction and subsequent sequencing. First, after the total RNA samples passed the quality test in this experiment, the mRNA was enriched using the NEBNext^®^ Poly (A) mRNA Magnetic Isolation Module, and a common transcriptome library was constructed using the NEBNext^®^ mRNA Library Prep Master Mix Set for Illumina, the insert size was 250 bp. Subsequently, PacBio P6 Binding Kit and PacBio C4 Sequencing Kit library sequencing reagents were used to load samples on the machine using magbeads, and data was collected for 1 × 240 minutes. After the qualified RNA (or polyA + RNA) is detected, the SMARTer PCR cDNA Synthesis Kit is used to synthesize the first cDNA strand. Once SMARTScribe Reverse Transcriptase is added to the 5′end of the mRNA, the terminal repair transferase will add a few extra base sequences to the 3′ end of the cDNA. These bases serve as binding points for oligonucleotide synthesis, extending the first strand of the cDNA to the end of the oligonucleotide, resulting in a second strand that facilitates synthesis of the cDNA strand and PCR amplification Double-stranded cDNA by universal primer sequences. Three generation sequencing builds a mixed library without screening fragments and >4 kb fragments. In our study, we combined PacBio Sequel and Illumina short-read sequencing to generate a more comprehensive full-length transcriptomes (Zhang W. M. et al., 2019). In addition, this approach enabled the transcriptional data to be more precisely correlated with the co-expression data of secondary metabolites produced and accumulated in suspension culture cells.

### Preprocessing of *de novo* Assembled Short Reads and SMRT Reads

The raw sequences of second-generation sequencing were cleaned by removing reads with an adapter, the ratio of *N* (*N* means base information cannot be determined) was greater than 10% of reads and low-quality reads (the number of bases with *Q*_*phred*_ ≤ 20 accounts for more than 50% of the reads) to get clean reads. And the raw reads of SMRT sequencing were preprocessed to trim out the low-quality data and the software SMRTlink v6.0 was used to filter and process the output. Then we get the Circular Consensus Sequence (CCS), also known as the reads of inserts (ROI). All ROI were further divided into full-length (FL) and non-full-length (nFL) transcriptional sequences according to whether 5′ primers, 3′ primers and poly-tail appeared at the same time. We subsequently adopted the following strategies to improve the accuracy of PacBio readings. First, we used the ICE algorithm to cluster the FLNC sequences of the same transcript to de-redundant to obtain the consensus sequence; then the obtained consensus sequence is corrected using ARROW software, so a polished consensus sequence was obtained for subsequent analysis. Secondly, in order to further improve the accuracy of the data, we corrected the polished consensus sequence of the second generation data through LoRDEC software with −k 21, −s 3, and default setting for other parameters, and analyzed the final polished consensus sequence (Salmela and Rivals, 2014; Zhang X. J. et al., 2019). Then, we used the ICE algorithm to perform isoform-level clustering on all sequences to obtain consensus sequences of isoform. Finally, we performed cluster deduplication on the corrected transcript sequences based on 95% similarity between the sequences, and used de-redundant sequences for subsequent analysis. These polished sequences were upload to NCBI with the TSA submission accession number of SUB7210106.

### Prediction of Csequences and Transcription Factors (TF)

The polished reads were used to predict the coding regions and the transcription factors before annotating. The ANGEL software was used to conduct the CDS prediction (Shimizu et al., 2006) and the iTAK software was carried out to do the prediction of transcription factors (Zheng et al., 2016).

### Functional Annotation

By applying the following software, the polished reads were further annotated to gain a database of unigenes with comprehensive analysis and annotation. Seven databases, including NCBI NR, NT, Swiss-Prot, Pfam, GO, KEGG and COG were blasted to do the annotation. The BLAST software with an *E*-value < 1e-5 was used in the NT database analysis. The Diamond BLASTX methods with an *E*-value < 1e-5 were analyzed in NR, COG, Swiss-Prot and KEGG annotations. The Hmmscan procedure was used in the Pfam database, and GO function categories were performed based on the Pfam database.

### LncRNA Prediction

We used CNCI (Sun et al., 2013), CPC (Kong et al., 2007), Pfam (Finn et al., 2016) and PLEK (Li et al., 2014) to predict the coding potential of transcripts obtained from CD-HIT de-redundancy.

### Simple Sequence Repeat Detection and Verification

Transcripts lengths over 500 bp were selected for mining Simple Sequence Repeat (SSR) markers by using the MISA (Beier et al., 2017), and the density distribution of different SSR types in transcripts was calculated. Then Primer3 was used to design SSR primers, which laid the foundation for the follow-up experiment (Untergasser et al., 2012). The search parameters were set as follows: the repetition times of mono-, di-, tri-, tetra-, penta-, and hexanucleotides nucleotides were at least 10, 6, 5, 5, 5, 5, respectively, and the distance between SSR sites and flanking sequences at both ends was greater than that of 50bp. SSR primers were designed using the following standard: (1) annealing temperature (*Tm*) from 57 to 63°C, the difference between forward and reverse primers was less than 2°C; (2) the primer size ranged from 18 to 27 bp, with an optimal size of 20 bp; (3) predicted product size range 100–300 bp; (4) three pairs of primers for each marker site, other parameters were set to default values.

We randomly selected 31 pairs of primers for verification in one type of suspension culture cells of *B. striata* by PAGE experiment after PCR amplification. The PCR reaction volume is 10 μL, including 15 ng template DNA, 6 μL 2 × PCR MIX, 0.75 μL each of the upper and lower primers, and 1 μL ddH_2_O. The amplification conditions were: 95°C pre-denaturation for 5 min; 95°C denaturation for 30 s, 58°C annealing for 30 s, 72°C extension for 60 s, 34 cycles, 72°C extension for 5 min. The amplified products were separated on a 10% polyacrylamide gel. The electrophoresis instrument is a PowerPac type steady state electrophoresis instrument. After electrophoresis at a constant pressure of 150 V for 90 min, the bands were observed and photographed after staining with silver nitrate.

## Results

The full-length transcripts expressed in suspension cultured callus were sequenced by Illumina and PacBio Sequel platform separately, which were combined to establish a database profiling throughout the whole growing period of the callus suspension culturing system of *B. striata*. The functional annotation and correlation analysis of the obtained unigenes were carried out.

### The Growth Curve of Callus Growing in Suspension Culture System

**FIGURE 1:**
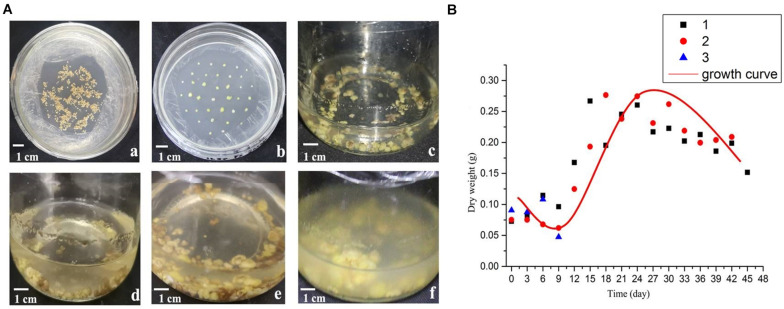
Morphology and growth curve of *B. striata* suspended cellus. **(A)** The growth state of *B. striata* cells in suspension culture system at different stages. **(a)** Induction culture for 45 days. **(b)** Subculture for 30 days. **(c)** Suspension culture for 3 days; **(d)** Suspension culture for 9 days; **(e)** Suspension culture for 15 days; **(f)** Suspension culture for 27 days. **(B)** The cell growth curve in suspension culture.

With the extension of culture time, the growth state of the suspension culture callus is shown in Figure 1A. The dry weight of the callus during culture was used to draw the growth curve (Figure 1B). As can be seen from the figure, the shape of the growth curve is shown as an “S” shape. The growth period is divided into four periods: lag period, exponential period, deceleration period and recession period. Among them, 0–9th days is the lag period, during which the dry weight of suspension cultured cells changes slowly, which is a process in which the cells gradually adapt to the environment; 9–18th days is the exponential phase, during which the cell growth rate gradually accelerates to the maximum; 18–24th days is the deceleration period, during which the cell growth rate gradually slows down; on the 24th day, the dry weight reached the maximum, then gradually decreases as the days increase.

### SMRT and Illumina Sequencing and Error Correction

By applying the PacBio Sequel sequencing platform, 1,015,753 polymerase reads were generated. After preprocessing, 54.02 Gb of subreads were obtained, and 902,688 CCS were obtained by the correction between subreads. 472,211 FLNC sequences were further classified from the CCS. In total, 246,933 consensus isoform sequences were finally obtained with SMRT Analysis software. Moreover, 49,768,628 to 77,860,762 paired-end raw data reads were generated from the Illumina HiSeq Sequencing 2000 platform, with GC content ranging from 44.73 to 47.38%. The sequence quality evaluation showed that Q20 was 97.44 to 97.95% and Q30 was 93.14 to 94.15% (Table 1). Finally, 589,522,461 clean reads were generated after quality filtering in an Illumina platform. These short reads were subsequently used to correct the consensus isoform sequences by PacBio sequencing. Among them, the average length of consensus is 2388 bp, which showed that the transcriptome sequencing result met the requirement for subsequent data assembling.

**TABLE 1:** Summary of quality of raw sequencing data based on Illumina sequencing.

Library ID	Sample name	Raw reads	Clean reads	Clean bases	Error rate (%)	Q20 (%)	Q30 (%)	GC content (%)
FRAS190050344-1a	BS01	56960062	55434040	8.32*G*	0.03	97.77	93.61	45.30
FRAS190050345-1a	BS02	64710036	63948232	9.59*G*	0.03	97.81	93.66	45.10
FRAS190050346-1a	BS03	62550314	60854658	9.13*G*	0.03	97.93	93.97	44.73
FRAS190050347-1a	BS04	68425960	67152294	10.07*G*	0.03	97.90	93.98	46.74
FRAS190050348-1a	BS05	77860762	73560574	11.03*G*	0.03	97.94	94.09	47.34
FRAS190050349-1a	BS06	67557700	66629280	9.99*G*	0.03	97.84	93.85	46.85
FRAS190050350-1a	BS07	62983310	61705188	9.26*G*	0.03	97.89	93.89	45.85
FRAS190050351-1a	BS08	60802994	58798382	8.82*G*	0.03	97.93	94.03	45.81
FRAS190050352-1a	BS09	68038342	65917640	9.89*G*	0.03	97.88	93.93	45.32
FRAS190050353-1a	BS10	83089632	80731038	12.11*G*	0.03	97.87	93.86	45.40
FRAS190050354-1a	BS11	77495368	75638624	11.35*G*	0.03	97.85	93.92	46.77
FRAS190050355-1a	BS12	73740596	72411246	10.86*G*	0.03	97.91	93.97	46.57
FRAS190050356-1a	BS13	65165150	63572558	9.54*G*	0.03	97.83	93.82	46.50
FRAS190050357-1a	BS14	55533042	54499344	8.17*G*	0.03	97.95	94.07	46.18
FRAS190050358-1a	BS15	59130306	58289554	8.74*G*	0.03	97.84	93.78	46.30
FRAS190050359-1a	BS16	53084272	52202430	7.83*G*	0.03	97.82	93.85	45.79
FRAS190050360-1a	BS17	60307100	59274226	8.89*G*	0.03	97.62	93.47	46.05
FRAS190050361-1a	BS18	49768628	48838162	7.33*G*	0.03	97.94	94.15	45.52
FRAS190050362-1a	BS19	50652868	49725090	7.46*G*	0.03	97.44	93.34	45.26
FRAS190050363-1a	BS20	52009780	51208802	7.68*G*	0.03	97.80	93.79	45.38
FRAS190050364-1a	BS21	54059774	53184004	7.98*G*	0.03	97.53	93.14	45.13
FRAS190050365-1a	BS22	53682236	52738402	7.91*G*	0.03	97.70	93.69	47.38
FRAS190050366-1a	BS23	56317392	55352946	8.3*G*	0.03	97.77	93.77	44.91

**FIGURE 2:**
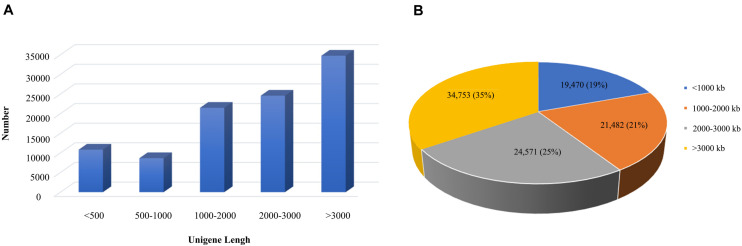
Distribution map of transcripts length of *B. striata*. The blue area represents the number of transcriptomes less than 1000 bp, the orange area represents the number of transcriptomes in 1000–2000 bp, the gray area represents the number of transcriptomes in 2000–3000 bp, and the yellow area represents the number of transcriptomes less than 1000.

According to the 95% similarity between the sequences, the corrected transcript sequences were clustered to remove redundancy, and the length and frequency distribution of transcripts before and after de-redundancy were counted, we finally obtained 100,276 transcripts. Among them, 19,470 transcripts (19%) with length less than 1000 bp, 21,482 transcripts (21%) had length of 1000–2000 bp, 24,571 transcripts (25%) had length of 2000–3000 bp and 34,753 transcripts (35%) with length more than 3000 bp. Thus, it could be seen that the transcript data were mainly more than 3000 bp, which is completely in line with the expected results of the third generation sequencing (Figure 2).

### Analysis of CDS and Transcription Factors of Unigenes in Transcriptional Group

**FIGURE 3:**
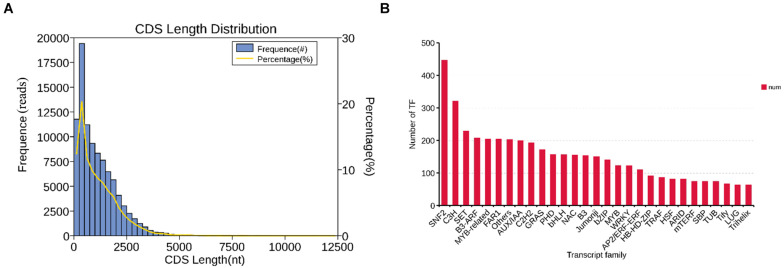
The distribution of CDS and TF. **(A)** CDS length distribution map of *B. striata* transcriptional group: Abscissa is the predicted length of CDS; ordinate is the number of transcripts of CDS. **(B)** TF analysis: *B. striata* transcriptional group TF analysis predicted that the above picture shows the top 30 transcription factor families with the largest number of annotated transcripts.

In the CDS prediction, a total of 95,302 coding sequences were predicted. Further statistics found that with the growth of predicted CDS, the number of unigenes showed a downward trend. The size of the fragments ranges from 48 to 3,767 nt, and there were 51,426 unigenes fragments in the range of 100–500 nt, with the highest proportion reaching 53.96%; while only 176 unigenes fragments were > 2000 nt, accounting for 0.18% (Figure 3A). In the prediction of plant transcription factors, a total of 5,585 transcription factors (TF) were annotated into 92 TF families. Among them, the SNF2 family is the most abundant TF family found in this study, with 446 transcription factors, followed by the C3H family with 321, and the SET family with 229 (Figure 3B).

### Functional Annotation and Classification

In order to obtain comprehensive gene function information, gene function annotations were carried out on the non-redundancy sequences by using CD-HIT software. The databases were used including Nr, Nt, Pfam, KOG/COG, Swiss-prot, KEGG, GO. The results showed that 86,075 (85.84%) transcripts were annotated in at least one database and 31,211 (31.13%) transcripts were annotated in all databases. 80,616 (80.39%) and 64,752 (64.57%) transcripts are annotated in Nr and Nt databases, respectively.

**FIGURE 4:**
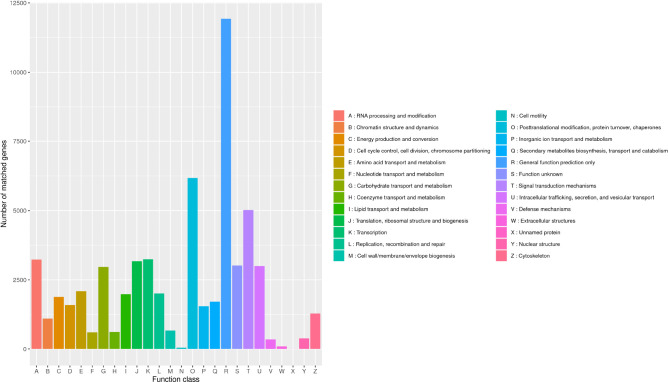
Classification of *B. striata* in KOG. The transcripts could be roughly divided into 26 categories according to their function (represented by A-Z), and the number of genes in each category was counted.

**FIGURE 5:**
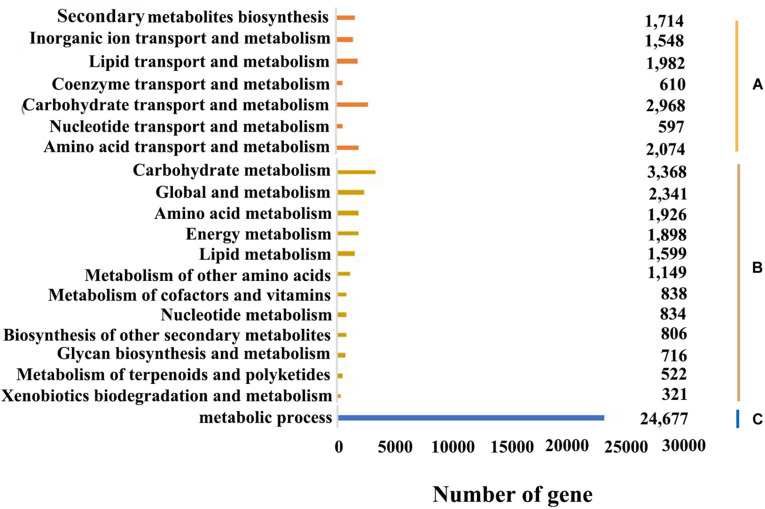
Functional annotation and classification of *B. striata*. **(A)** Metabolism system process (KOG annotation). **(B)** Metabolism system pathway (KEGG annotation). **(C)** Metabolism system process in *B. striata* (GO functional classification).

In the KOG database, there were 53,316 (53.17%) transcripts that were similar to the genes in the database. The transcripts could be roughly divided into 26 categories according to their function (represented by A–Z in Figure 4), and the number of genes in each category was counted. As can be seen from the picture, the KOG function of genes was relatively comprehensive, involving most life activities, and the number of genes related to general function prediction was the largest, with the number of 11,924. On the contrary, the number of genes related to cell motility only had 47, and the expression abundance of other kinds of transcripts was different. Among them, the number of genes related to metabolic function had 11,513 (Figure 5A).

Overall, 52,018 transcripts were assigned into the three major categories: biological process, molecular function and cellular component (Figure 5C). And 543,568, 91,610, and 165,997 genes were involved in these three categories, respectively. Since there may be multiple annotations for the same transcript, the total number of transcripts annotated to the GO database was greater than the number of transcripts actually annotated. And in biological process, metabolic process, cellular process and single-organism process involved the most genes, while in molecular function were binding and catalytic activity, and in cellular component were cell, cell part and organelle.

The KEGG database can systematically analyze the metabolic pathways of genes in cells. By applying KEGG clustering, 80,021 transcripts were annotated in 47 pathways in total. These transcripts were clustered into 363 metabolic pathways, including carbohydrate metabolism, amino acid metabolism, metabolism of terpenoids and polyketides, lipid metabolism, translation, transcription, etc. (Figure 5B). Among them, the number of genes involved in metabolic pathways was the largest, with 16,318, accounting for 20.23% of the total, of which genes for carbohydrate metabolism account for 20.64% of metabolic pathway genes; followed by genetic information, with 9,081, accounting for 11.26%. And the number of genes participated in cellular processes was 5,223, accounting for 6.48%; the number of organic systems was 7,048, accounting for 8.74%; while the number of environmental information processing was the least, with 4,249 genes involved, accounting for 5.27% of the total.

### LncRNA Analysis

**FIGURE 6:**
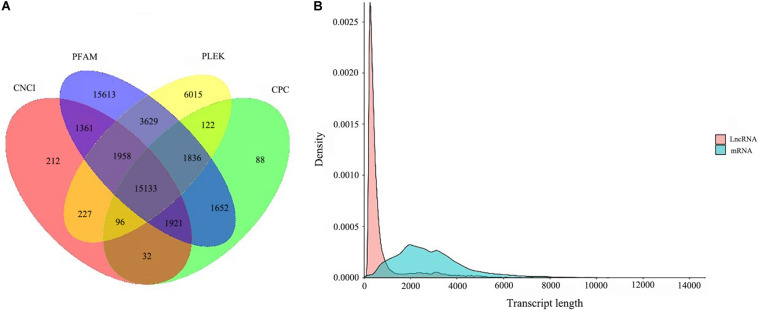
LncRNA analysis. **(A)** coding potential prediction Venn diagram. The sum of the numbers in each large circle represents the number of Lnc predicted by the coding potential prediction software, and the overlapping part of the circle represents the common number of Lnc between combinations. **(B)** and the length distribution of LncRNA and mRNA.

A total of 15,133 lncRNA transcripts predicted by CPC, CNCI, pfam protein domain analysis and PLEK were shown in Figure 6A. After removing the transcripts of coding potential (score > 0), PLEK and CNCI retained 29,016 and 20,940 transcripts from the predicted 100,276 full-length unigenes with the length of over 200 nt, respectively. After filtering by CPC, a total of 20,880 unigenes transcripts were predicted for further analysis. Finally, a total of 15,133 transcripts were identified as predictive lncRNA. According to the statistics of length distribution, the length of these lncRNA was almost smaller than that of 4000 nt (96.96%), and the length range from 200 to 1000 nt was the richest distribution of lncRNA (Figure 6B).

### SSR Analysis

**FIGURE 7:**
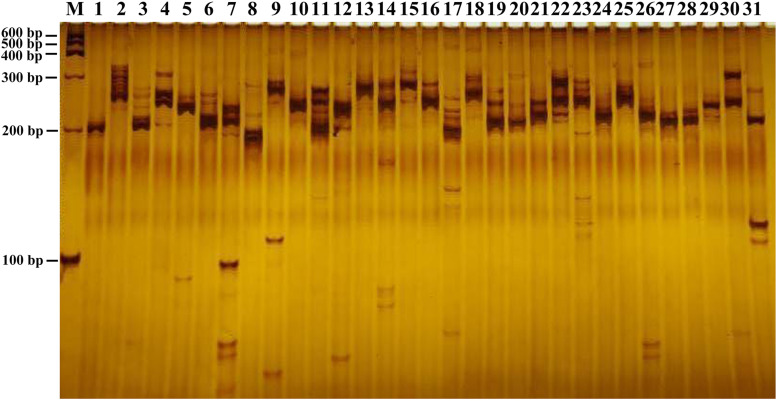
SSR validation of *B. striata*. The 31 pairs of primers were amplified.

In this study, a total of 168,965 SSRs sites were detected from 68,996 transcripts of the formed database by MISA, of which 22,393 contained more than one SSR. The SSRs detection frequency (the percentage of the total transcripts number of SSRs to the total number of detection sequences) was 68.81%, with an average of one SSR site per 4.35 kp (the ratio of the total length of the detection sequence to the total number of SSR sites). It was shown that the formed database was rich in microsatellite repeat types. Among them, single nucleotide repeat was the main repeat type, accounting for 81.54%, followed by dinucleotide and trinucleotide, accounting for 11.35 and 6.86%, respectively, and 29,124 transcripts existed as a complex repeat type (Table 2). Then we used Primer3 to design SSR primers, and a total of 103,490 pairs of primers were obtained (Additional file 1). Finally, 31 pairs of primers were randomly selected to amplify the genomic DNA of 1 strain of *B. striata*. Each pair of primers can be amplified and stabilized (Figure 7). Therefore, SSR primers in this study can provide corresponding data for subsequent validation.

**TABLE 2:** SSR repeat types and numbers.

Number	Repeat (5–8)	Repeat (9–12)	Repeat (13–16)	Repeat (17–20)	Repeat (21–24)	Main repeat motif
Mono-	0	31310	23491	38424	44555	A/T (96.06%)
Di-	8868	5288	2576	1541	903	TC/TA (35.14%)
Tri-	10666	812	95	14	5	ATG/CAA (10.29%)
Tetra-	200	0	0	0	0	GCCC/TTTA (16.42%)
Penta-	63	0	0	1	0	CTCTC/TTTCT (35.71%)
Hexa-	153	0	0	0	0	

## Discussion

*Bletilla striata* is a traditional and precious Chinese herbal medicine, which not only has high medical value, but also has good ornamental value. In recent years, with the demand of the market, the disorderly mining activities have increased, and the plant ecosystem has been seriously damaged gradually. Therefore, tissue culture of *B. striata* can quickly propagate a large number of seedlings to maintain the ecological environment and meet the needs of artificial cultivation. Compared with well-researched plants, although there is a database of nucleotides related to *B. striata* roots, stems and leaves (Xu et al., 2018), there is no related transcriptome study on tissue cultured *B. striata*. Therefore, it is necessary to study the transcriptome of tissue cultured *B. striata*, in order to lay the foundation for the study of the genetic mechanism of cell growth and development and the accumulation of secondary metabolites. Full-length cDNA sequences are a fundamental resource for genomic research. Thus, in this study, SMRT and Illumina sequencing were used to sequence the transcriptome of *B. striata* suspension culture cells, and the first nucleotide database of the *B. striata* suspension culture system was established. It provided the complete transcriptome data of *B. striata* for subsequent research, and provided an important theoretical basis for the effective development and utilization of *B. striata* resources.

The study of structural and functional genomics is the basis for understanding plant metabolism. In order to carry out such research, it is essential to obtain high-quality genomic and transcriptional sequence. Based on RNA-seq data, we found that *B. striata* contains a large number of almost the same homologous sequences, indicating that its genome is very large. SMRT can produce a sequence reading of kilobase (Sharon et al., 2013). The NGS technology provides a low-cost, labor-saving and rapid transcriptome sequencing and identification method (Lo and Shaw, 2019), which makes it possible to study various functional genomes of organisms. Therefore, we propose to use SMRT technology and SGS technology to obtain comprehensive and high-quality of *B. striata* full-length transcripts.

**FIGURE 8:**
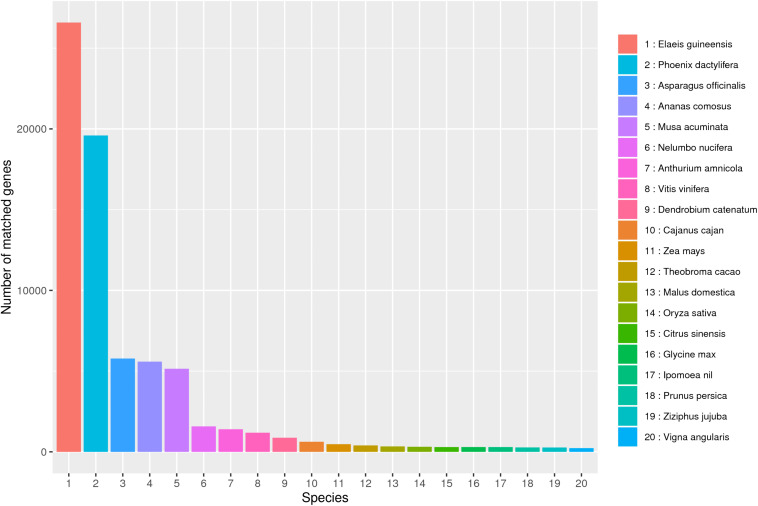
NR database species annotation. A total of 20 species were compared.

In this study, 57.54 G Polymeraseread data were obtained after SMRT sequencing, including 902,688 CCS sequences and 472,211 FLNC sequences. After correcting by the SGS and removing the redundant sequence, 100,276 transcripts were obtained. Based on these high-quality transcripts, a series of annotation analyses was carried out. NR analysis showed that *B. striata* is highly similar to three major species (*Elaeis guineensis*, *Phoenix dactylifera*, and *Asparagus officinalis*), which provided a reference plant for the study of *B. striata* (Figure 8).

Gene functional annotation and KEGG pathway analyses are helpful to predict potential genes and their functions at the genome-wide level. In the *B. striata* transcriptome, the main GO functional clusters were involved in cellular processes and metabolic processes in biological functional categories, cells and cellular parts in cell composition categories, and catalytic activity and binding categories in molecular functional categories. The results showed that the transcription of *B. striata* was related to the above functions. *Houttuynia cordata* (Wei et al., 2014), *Drynaria roosii* (Sun et al., 2018), and *Crocus sativus* (Qian et al., 2019) had similar results. However, in the short-leaf *Carex* transcriptome, these sequences were mainly involved in proline transport and chlorophyll biosynthesis in biological process (Teng et al., 2019). This showed that there were significant differences between different kinds of plants. The results of KOG annotations showed that there were 1,714 transcripts annotated as secondary metabolites for synthesis, transportation and metabolism, and 2,094 transcripts annotated as amino acid transportation and metabolism, indicating that the secondary metabolism and amino acid metabolism of suspended cells in *B. striata* suspension culture systems were more active. It laid a foundation for promoting the growth of *B. striata* suspended cells. In this study, a large number of *B. striata* transcripts were obtained at the transcriptome level for the first time, which provided valuable sequence information for screening new functional genes or studying molecular mechanisms.

KEGG analysis showed that 80,021 transcripts were involved in 363 known metabolic or signal pathways, including plant hormone signal transduction, carbohydrate metabolism, terpenoid metabolism, phenylalanine metabolism, flavonoid metabolism, etc. *B. striata* is an important medicinal plant, which is rich in secondary metabolites and is an important target for genome research. In this study, the number of genes involved in metabolic pathways was the largest, of which 3,368 transcripts were involved in carbohydrate metabolism, and 806 transcripts were related to the biosynthesis of secondary metabolites (Figure 5B). It showed that during the development of *B. striata* suspended cells, in addition to the most basic life activities such as replication, translation and transcription, carbohydrate metabolism and the secondary synthesis of metabolites played an important role in the formation and development of *B. striata* callus. These results have a certain reference value for the further study of gene function in the future.

LncRNAs, a novel class of non-protein coding transcripts longer than 200 nt, are key regulatory molecules that can regulate gene expression at many different levels (Di et al., 2014; Jia et al., 2018). Most of the annotated lncRNA are transcribed by RNA polymerase II (PolII). Therefore, they are structurally similar to mRNA and may have cap structure and polyA (Zhang X. P. et al., 2019). However, by using NGS, most studies aimed at identifying lncRNA will inevitably lack polyA, thus affecting the accuracy of the data (Zhang et al., 2014). At present, more and more studies focus on the function of plants, such as *Astragalus* (Li J. et al., 2017), *Salvia miltiorrhiza* (Xu et al., 2015), etc., which facilitated to the exploration of the biosynthesis of plant active components. However, compared with animals, the function of plant lncRNA is still unclear (Chen et al., 2019). In this study, 15,133 lncRNA transcripts were identified by four analytical methods. The length of the obtained lncRNAs was shorter than that of the protein-coding mRNA, and its most abundant length ranges from 200 to 1000 nt, which is consistent with previous studies (Xu et al., 2017). lncRNA played an important role in stress response, cell cycle regulation, etc., indicating that it might affect the growth and development of *B. striata* suspended cells. Therefore, we need to further study their role in *B. striata*.

Simple Sequence Repeat markers play an important role in genetic diversity research, population genetics, linkage map, comparative genomics and association analysis (Taheri et al., 2018; Yang et al., 2018). In this study, we identified 68,996 transcripts from *B. striata* database. The results showed that mono- repeats was the most abundant type, followed by di- and tri- repeats, which was consistent with the previous report (Xu et al., 2018). And we amplified 31 pairs of primers selected at random and obtained stable amplified bands. Therefore, in the future research, based on some of our current genome resources, more SSR markers will be generated and can be used for genotyping and phenotypic typing, which provides a theoretical basis for genetics and breeding of *B. striata*.

We have produced a total of 100,276 full-length transcripts, 95,302 CDS, 68,996 SSRs and 15,133 lncRNA. Through the analysis of NGS data, the regulation mechanism of this plant on growth and development was clarified. In this study, SMRT sequencing method was used for the first time to provide a complete full-length transcriptome. The resulting transcriptome map will provide convenience for future functional genomics research and provide a basis for further screening and genetic engineering breeding.

## Data Availability Statement

These polished 196 sequences were upload to NCBI with the TSA submission number of SUB7210106.

## Author Contributions

DX, MW, and JS conceived the study. LL, HL, WW, CH, XL, and SX performed the experiments and carried out the analysis. DX, LL, HL, and WW designed the experiments and wrote the manuscript. All authors read and approved the final manuscript.

## Conflict of Interest

The authors declare that the research was conducted in the absence of any commercial or financial relationships that could be construed as a potential conflict of interest.

## Supplementary Material

Additional File 1 The SSR primers. For each SSR site, three primer pairs were designed.
