# Erratum: Full Transcriptome Analysis of Callus Suspension Culture System of *Bletilla striata*

**DOI:** 10.3389/fgene.2020.636385

**Published:** 2021-02-11

**Authors:** Frontiers Production Office

**Affiliations:** Frontiers Media SA, Lausanne, Switzerland

**Keywords:** *Bletilla striata*, suspension culture, transcriptome sequencing, functional annotation, lncRNA, SSR

Due to a production error, there was a mistake in Figure 4 as published. The published Figure 4 was the same as Figure 8. The correct Figure 4 appears below.

**Figure 4:**
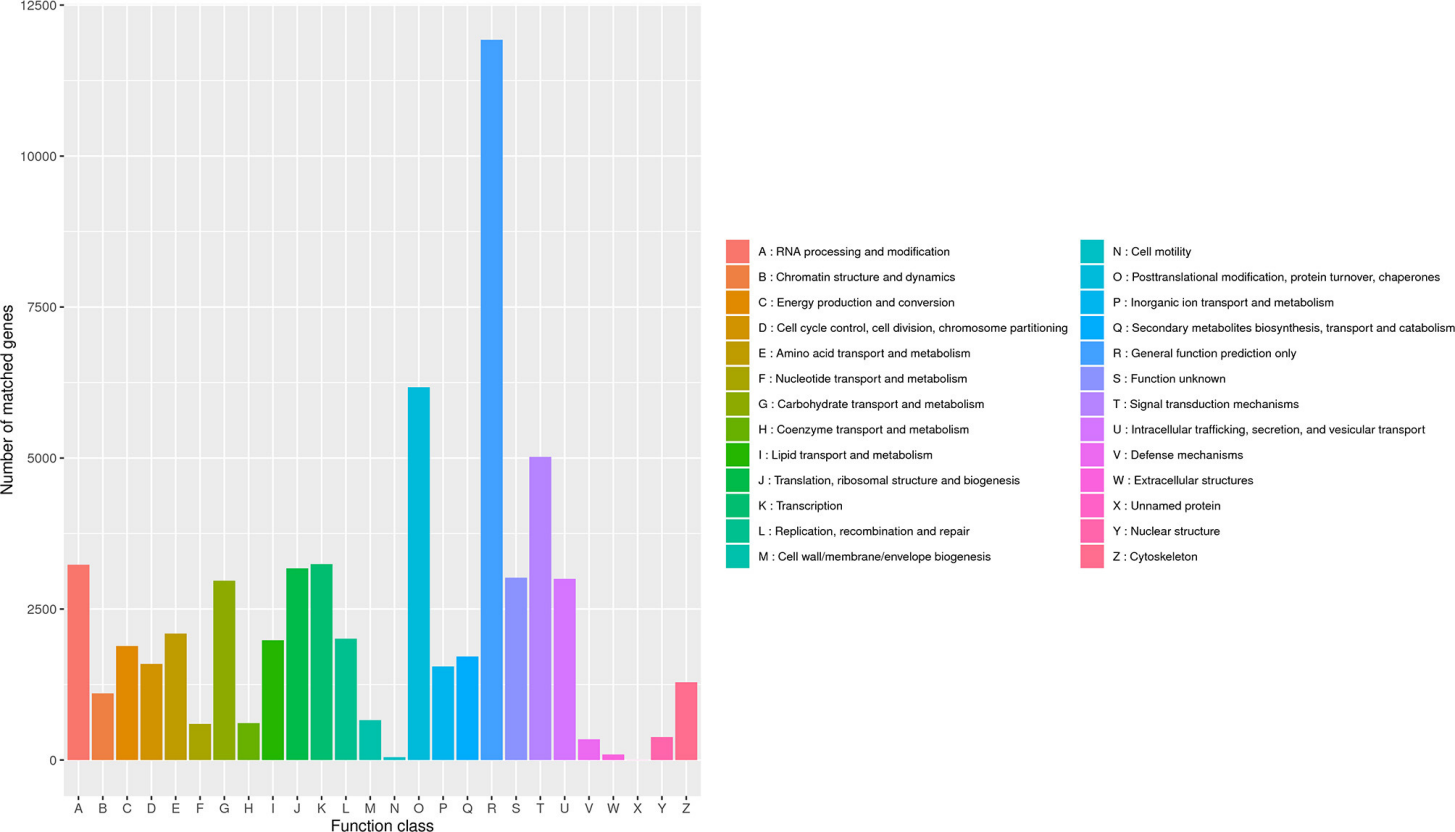
Classification of *B. striata* in KOG. The transcripts could be roughly divided into 26 categories according to their function (represented by A-Z), and the number of genes in each category was counted.

The publisher apologizes for this mistake. The original article has been updated.

